# Prognostic significance of E-cadherin expression in prostatic carcinoma: A protocol for systematic review and meta-analysis

**DOI:** 10.1097/MD.0000000000019707

**Published:** 2020-04-10

**Authors:** Xiwen Zhang, Zhenhua Zhang, Shuntai Chen, Juling Jiang, Runzhi Qi, Xue Mi, Xing Zhang, Yupeng Xi, Honggang Zheng, Baojin Hua

**Affiliations:** a Department of Oncology, Guang’anmen Hospital, China Academy of Chinese Medical; b Beijing University of Chinese Medicine, Beijing; c Shanxi University of Chinese Medicine, Xianyang, Shanxi Province, China.

**Keywords:** E-cadherin, meta-analysis, prognosis, prostatic carcinoma, protocol

## Abstract

**Background:**

Increasing studies were performed to explore the prognostic value of E-cadherin in prostatic carcinoma, however, with inconsistent results. Hence, this systematic review is aimed to evaluate the prognostic role of E-cadherin in patients with prostatic carcinoma (PCa).

**Methods:**

A comprehensive literature search in all available databases will be conducted to identify eligible studies. We will employ hazard ratios (HRs) and 95% confidence intervals (95% CIs) to estimate the correlations between E-cadherin expression and overall survival (OS), disease-free survival (DFS), relapse-free survival (RFS), progression-free survival (PFS) and clinicopathological features. Meta-analysis will be performed using Review Manager (Revman) 5.3.5 software (Cochrane Community, London, United Kingdom) and STATA 14 software (version 14.0; Stata Corp, College Station, TX).

**Results:**

This study will provide a high-quality synthesis of current evidence of the correlations between snail expression and OS, DFS/RFS, PFS and clinicopathological features.

**Conclusion:**

The study will provide updated evidence to assess whether the expression of E-cadherin is in association with poor prognosis in patients with PCa.

**Ethics and dissemination:**

It is not necessary for ethical approval because individuals cannot be identified. The protocol will be disseminated in a peer-reviewed journal or presented at a relevant conference.

**Prospero registration number:**

This systematic review protocol has been registered in the PROSPERO network (No. CRD42019128353).

## 1 Introduction

Prostate cancer (PCa) is rank as the second most common carcinoma and the fifth leading cause of cancer death among men worldwide. In 2018, approximately 1.3 million new cases of PCa will be diagnosed and an estimated 359,000 deaths occurred in the world. It is the most commonly diagnosed cancer in men in more than half of the world's countries.^[1]^ In the past 15 years, the incidence of PCa in China and Asia has increased rapidly.^[2]^ The factors leading to an increase in prostate cancer in China are not fully understood. However, they may include improvements in the progressive implementation of prostate-specific antigen screening and biopsy techniques or the impact of an increasingly westernized lifestyle.^[1]^

Compared with other types of cancer, PCa patients have a high 5-year survival rate in the past 15 years.^[3]^ Organ-confined PCa can be effectively treated via radical prostatectomy.^[4]^ However, in patients with clinically detectable metastases, androgen deprivation therapy is considered as a first-line treatment.^[5]^ Once hormone resistance develops, for advanced PCa, tumor invasion and metastasis are life-threatening events.^[6]^

Prostate cancer has a wide range of metastatic sites. However, boneis the most frequent site for metastasis, and distant lymph nodes, lungs, and liver are also the common sites of metastasis.^[7]^

Various steps are involved in tumor metastasis, including the epithelial-to-mesenchymal transition (EMT) process, whereby epithelial cells lose their ability to adhere to adjacent cells and extracellular matrix proteins and acquire mesenchymal phenotype. EMT is characterized by down-regulation of the epithelial marker E-cadherin, and up-regulation of interstitial markers such as vimentin, N-cadherin, and snails.^[8]^

The transmembrane protein E-cadherin is one of the key molecules that form adhesive intercellular connections between epithelial cells and establishes cell polarity, so it is considered a hallmark of epithelial state.^9^,^10^ Loss of E-cadherin expression is considered to be a hallmark of EMT, while a decrease in E-cadherin expression occurs during the development of PCa, such as migration, invasion, and eventual metastasis.

Besides, recent evidence suggests that the down-regulation of E-cadherin enhances PCa chemoresistance and affects the patient's prognosis.^11^,^12^However, a recent study demonstrated that the quintessential epithelial marker E-cadherin promotes metastasis of invasive ductal breast carcinoma by enhancing the survival of tumor cells. Moreover, it suggested that the functional impact of E-cadherin in metastasis is not likely to be just a side product of its association with the epithelial status.^[13]^

Therefore, the role of E-cadherin in the prognosis of the tumor still needs further validation. Given that the results are controversial, we aim to systematically evaluate the prognostic role of E-cadherin in PCa patients.

## 2 Methods

### 2.1 Study registration

The meta-analysis protocol will be developed following the Preferred Reporting Items for Systematic Reviews and Meta-Analysis Protocols (PRISMA-P) statement guidelines.^[14]^ The PRISMA-P checklist for the protocol is provided in the PRISMA-P-checklist.

The protocol of the systematic review has been registered on PROSPERO. The registration number is CRD42019128353.

### 2.2 Data sources and search strategy

#### 2.2.1 Electronic searches

Medline, EMBASE, Cochrane Central Register of Controlled Trials (CENTRAL), China National Knowledge Infrastructure Database (CNKI), Chinese Scientific Periodical Database (VIP Database) and Wanfang Database will be our electronic databases for retrieval. The period of the electronic database search is from inception to May 2019.

The strategy will be created based on a discussion among all reviewers according to the Cochrane handbook guidelines. The following search terms will be used: “prostate”, “tumor”, “cancer”, “neoplasm”, “E-cadherin”, “E-CAD”, “cadherin-1”, “prognostic”, and “survival”

The preliminary search strategy in Table 1 will be used for Medline. This search strategy will be modified and adapted to other databases based on their specific requirements.

**Table 1:**
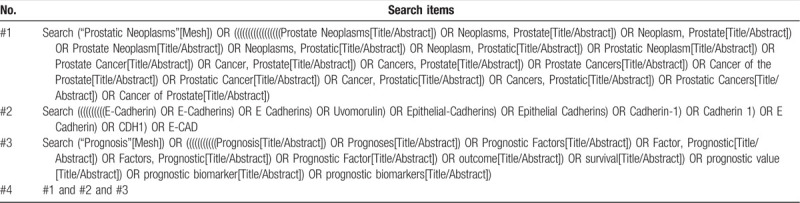
Preliminary search strategy for Medline.

#### 2.2.2 Searching other resources

In addition to the electronic databases, the library of the Chinese Academy of traditional Chinese medicine and Peking Union Medical College will also manually search for printed journals and related textbooks. Besides, a reference list of identified articles that will be obtained from the original search will be manually searched to identify other relevant studies.

### 2.3 Inclusion criteria for study selection

The included articles must meet the following inclusion criteria:

(1) patients diagnosed with prostate cancer using pathological and histological examinations;
(2) E-cadherin expression detected in primary tumor tissues;
(3) full text, original Chinese and English Research papers;
(4) Statistical results, including hazard ratios (HRs) and 95% confidence intervals (CIs) directly reported or calculated from demographic data or survival curves;
(5) independent E-cadherin expression level data.

If duplicate data from other articles appear, select only studies with more details and larger sample size. Comments, letters, meeting abstracts and reviews are not included.

### 2.4 Data collection and analysis

#### 2.4.1 Selection of studies

**Figure 1:**
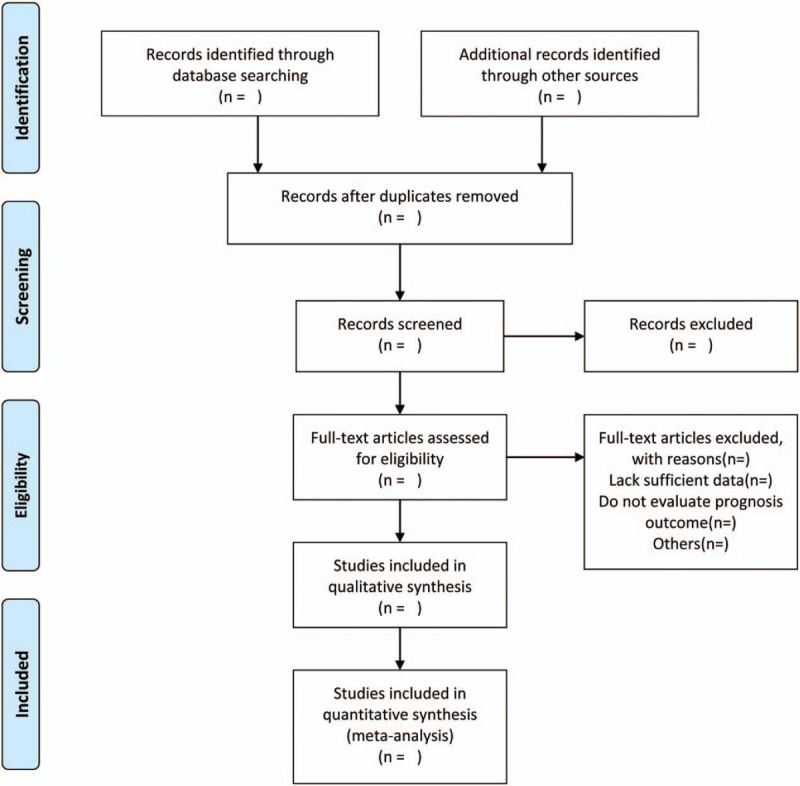
Flow diagram of studies search and selection.

To ensure the accuracy of the included studies, all review authors completed good training and adhered to the process summarized according to the PRISMA flow diagram (Fig. 1). The records from the electronic databases and other resources will be uploaded into a database created by Endnote X9, which will allow for systematic storage of the records as well as the ability to remove duplicate articles. The titles, abstracts, and keywords of all retrieved records will be independently screened by 2 review authors. Studies that meet the criteria will be further determined for inclusion by reading the full text. Those trials seemingly meeting inclusion will be obtained for further assessment of full texts by the same 2 reviewers. Excluded studies and the reasons for exclusion will be recorded. Disagreements between the 2 reviewers will be resolved by consensus or by involving a third independent an arbiter.

#### 2.4.2 Data extraction and management

The details of the eligible studies will independently record by 2 review authors, including the first author, publication year, characteristics of patients, pathology type, E-cadherin assay methods, total cases, clinical-pathological features, and outcomes.

The value of HRs and the 95% CI describing continuous outcomes will be obtained directly in some studies. For studies whose HRs and 95% CI were not reported in included studies directly, we will calculate or estimate them according to the method of Tierney from survival curves which were constructed using the Kaplan-Meier method and log-rank test.^15^,^16^ If there are discrepancies between the two reviewers, a final consensus will be reached by discussion or an arbiter.

### 2.5 Assessment of quality in included studies

The quality of each selected study will be independently assessed by two investigators using the Newcastle-Ottawa Quality Assessment Scale (NOS) which has been recognized as a validated quality assessment instrument for nonrandomized trials that assesses 3 parameters of study quality: selection, comparability, and outcome assessment.^[17]^ Each study with at least scores 5 is considered as high quality.^[18]^ The discrepancy of quality assessment among the investigators will be resolved through discussion or reached the consensus by an arbiter.

### 2.6 Measures of prognosis

The overall survival (OS), disease-free survival (DFS)/ relapse-free survival (RFS), and progression-free survival (PFS) will be taken as prognostic outcomes. The results will be expressed as HRs with 95% CIs.

### 2.7 Management of missing data

If the inadequate and missing data are detected involved in trials, the reviewers will try to contact the corresponding author via email integrate the data. If the data are not obtained, we will analyze the currently available data only and will discuss its potential effects.

### 2.8 Assessment of heterogeneity

The heterogeneity of all studies will be checked by Cochran's Q test and Higgins I^2^ statistic.^[19]^ Generally, the *I* ^*2*^ >75%, 50%–75%, 25%–50%, and <25% respectively correspond to the extreme, high, moderate and low heterogeneity. The threshold of significant heterogeneity will be set as *I* ^2^ > 50% in this study.

### 2.9 Assessment of publication biases

If there are a sufficient number of articles greater than 10, we will assess the publication bias by a funnel plot. Funnel plot visual inspection and statistical tests (Begg and Egger tests)^[20]^ will be used to evaluate publication bias in the study.

### 2.10 Statistical analysis

Review Manager (Revman) 5.3.5 software (Cochrane Community, London, United Kingdom) and STATA 14 software (version 14.0; Stata Corp, College Station, TX) will be used for data analysis and synthesis when a meta-analysis is allowed. The fixed-effect model will be used for pooling homogeneous data (I^2^ < 50%). Otherwise, a random effect model is used to analyze (I^2^ ≥ 50%).^21^,^22^ If substantial heterogeneity is identified, we will explore the possible causes of sensitivity analysis and subgroup analyses.

### 2.11 Subgroup analysis

Subgroup analysis will be conducted to identify possible sources of heterogeneity. Different ethnicities, countries and statistical analyses will be considered as subgroup analysis.

### 2.12 Sensitivity analysis

Reanalyzing the data using different statistical methods, mainly sequential omitting individual studies to test the stability of the pooled results, will be utilized for the sensitivity analysis of the meta-analysis.

## 3 Discussion

PCa is the most common neoplasm of males and despite the progressive decline in its incidence and mortality, it is still the second common cause of cancer-related death among men.^[1]^ The National Comprehensive Cancer Network (NCCN) guidelines defined the high-risk localized prostate cancer as initial PSA >20 ng/ml, clinical-stage ≥T3a and Gleason score ≥8.^[23]^ However, these clinical markers do not adequately discriminate between Localized tumors and those that will progress to be metastatic, so efforts are now directed towards using a combination of biological rather than clinical markers to predict prognosis and response to therapy.^[24]^

Several studies have shown that EMT transcription factor E-cadherin plays a key role in metastasis and drug resistance of prostatic carcinoma. Loss or aberrant expression of E-cadherin is related to PC progression, metastasis, and poor prognosis through two different mechanisms, cell-cell adhesion, and paracrine action.^[25]^ However, the results of this researches are controversial. Recent research implicates that although lost E-cadherin is sufficient to introduce oncogenic transformation in prostatic epithelia, it also induces cell apoptosis and disrupts epithelial structure, preventing atypical prostatic intraepithelial neoplasia cells from progressing to tumor cells.^[26]^ Hence, we hope this review will provide more accurate and objective shreds of evidence of the relationship between the E-cadherin and the prognosis of patients with PCa. The findings will be published in a peer-reviewed journal.

There are some limitations to this review. Because of the barrier of language, only studies published in English and Chinese will be included, which may run the risk of heterogeneity. Also, the methods and cut-off definitions for evaluating E-cadherin expression may be different.

## Author contributions

Xiwen Zhang and Zhenhua Zhang contributed to the conception of the study. Xiwen Zhang, Shuntai Chen and Juling Jiang wrote the draft of manuscript, and was revised by Xing Zhang, Yupeng Xi and Honggang Zheng. The search strategy was developed by all of the authors. Xiwen Zhang, Xue Mi and Runzhi Qi will search, extract data, assess the risk of bias, and complete the data synthesis. Baojin Hua will arbitrate in case of disagreement and ensure the absence of errors. All authors approved the publication of the protocol.

**Conceptualization:** Xiwen Zhang, Zhenhua Zhang.

**Data curation:** Xiwen Zhang, Xue Mi, Runzhi Qi.

**Formal analysis:** Xiwen Zhang, Xue Mi, Runzhi Qi.

**Investigation:** Xue Mi, Runzhi Qi.

**Methodology:** Juling Jiang.

**Project administration:** Baojin Hua.

**Writing – original draft:** Xiwen Zhang, Shuntai Chen and Juling Jiang.

**Writing – review & editing:** Xing Zhang, Yupeng Xi, Honggang Zheng.
